# Case Report—An Unusual Presentation of Cervical Meningioma as a Carotid Body Mass Diagnosed by Fine Needle Aspiration Cytology

**DOI:** 10.1111/cyt.70018

**Published:** 2025-08-21

**Authors:** Poorva Singh, Liam Chen, Zuzan Cayci, Khalid Amin

**Affiliations:** ^1^ Department of Laboratory Medicine and Pathology University of Minnesota Minneapolis Minnesota USA; ^2^ Department of Radiology and Nuclear Medicine University of Minnesota Minneapolis Minnesota USA

**Keywords:** carotid body, cytology, FNA, meningioma, paraganglioma

## Abstract

We describe an unusual case of cervical meningioma presenting as a carotid body tumour. This case emphasises the importance of cytoradiologic correlation in lesions with atypical imaging findings; and the crucial role of fine needle aspiration cytology in accurately diagnosing masses in precarious anatomic sites of the head and neck.

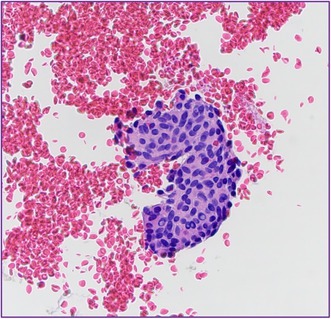

## 1 Introduction

Masses near the carotid artery bifurcation are rare occurrences and are commonly diagnosed as paragangliomas, schwannomas, enlarged lymph nodes, and rarely meningiomas. They are evaluated using ultrasound with Doppler, MRI (Magnetic Resonance Imaging), Computed Tomography (CT) or angiography, followed by imaging‐guided FNA (Fine Needle Aspiration) or core biopsy for tissue diagnosis. We present the fascinating case of a carotid body mass considered to be a paraganglioma with unusual imaging that was diagnosed as a meningioma on cytopathology, highlighting the significance of cytologic diagnosis in cases with atypical imaging findings.

## 2 Case Presentation

Our patient is a 48‐year‐old woman who presented with a long‐standing, slowly growing mass in the right side of her neck for 14 years. It was not initially associated with pain or other symptoms, but with its increasing size, she developed noticeable hoarseness of speech, occasional choking with ingestion of solids and liquids, and the feeling of something stuck in her throat. She denied difficulty breathing, facial flushing, or sweating. Clinical examination found ipsilateral vocal cord paresis causing hypernasal speech, neuropathy of the ipsilateral vagus and hypoglossal nerves, mass effect on the larynx, and pharyngeal narrowing. She also suffered from comorbidities including Type II Diabetes Mellitus, hypertension, obesity, and severe anxiety with frequent panic attacks.

**FIGURE 1:**
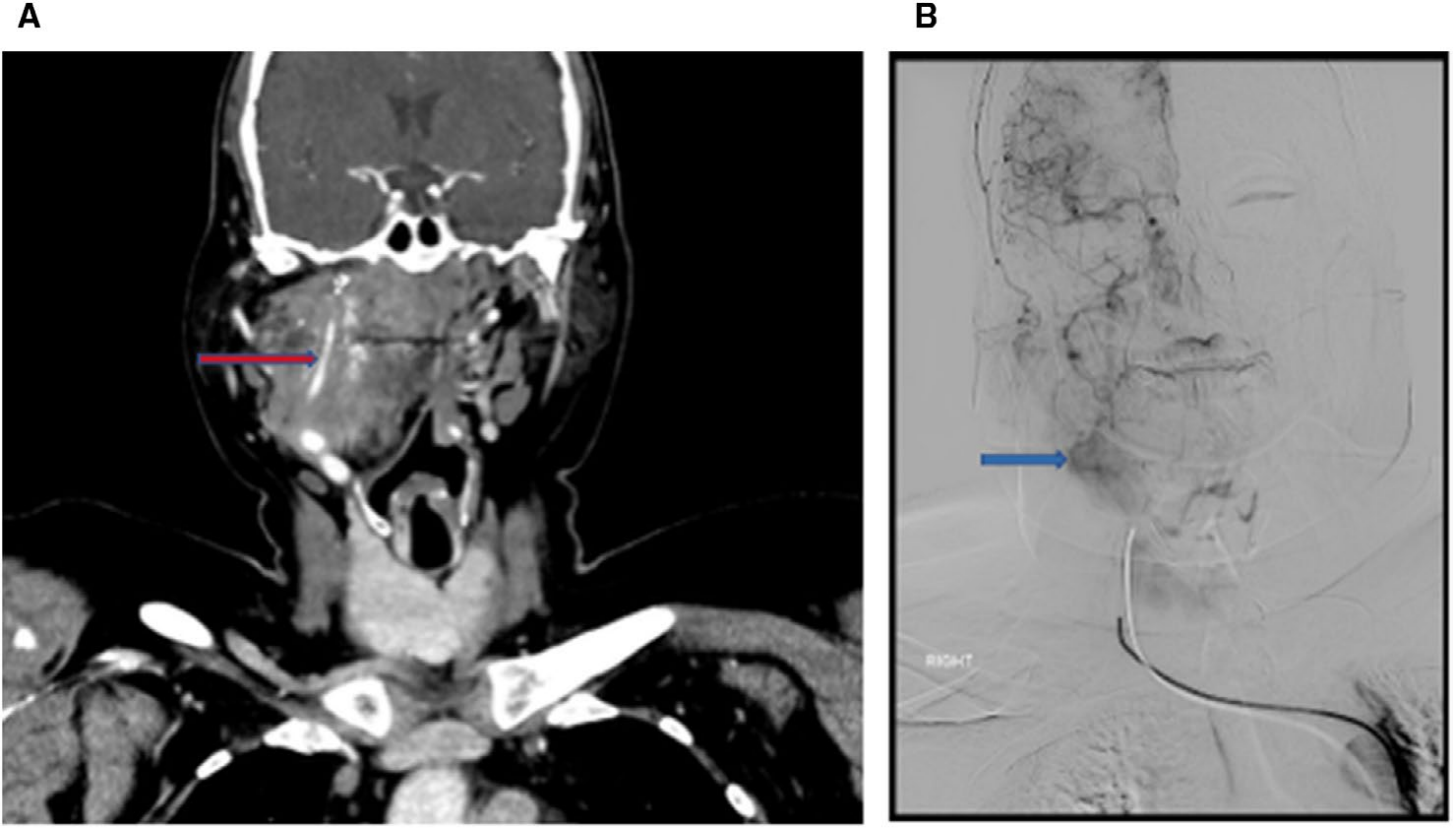
(A) CECT demonstrating a right carotid space mass causing smooth narrowing of the Internal Carotid Artery (blue arrow), showing subtle diffuse enhancement and linear areas of more intense, vascular type enhancement in the center; (B) DSA showing tumour blush in the late capillary phase (blue arrow) in the expected region of the mass.

CT of the neck with contrast was performed, revealing an ill‐defined, heterogeneous mass in the right carotid space measuring 4.5 × 3.5 cm. The mass showed areas of central, vascular‐type contrast enhancement with slight increase in diffuse enhancement on delayed imaging (Figure 1A). There was smooth narrowing of the internal carotid artery and coarse calcifications at the cranial aspect of the mass, immediately below the skull base. The lesion encased but did not splay the carotid bifurcation and did not have any vascular pedicles feeding into it. Digital Subtraction Angiography (DSA) did not show early arterial enhancement but rather a tumour blush in the late capillary phase, which stayed on until the venous phase (Figure 1B). These features raised the possibility of a meningioma, schwannoma, or paraganglioma. MRI was deemed necessary to further define the lesion, but it was not possible due to the patient's severe claustrophobia.

**FIGURE 2:**
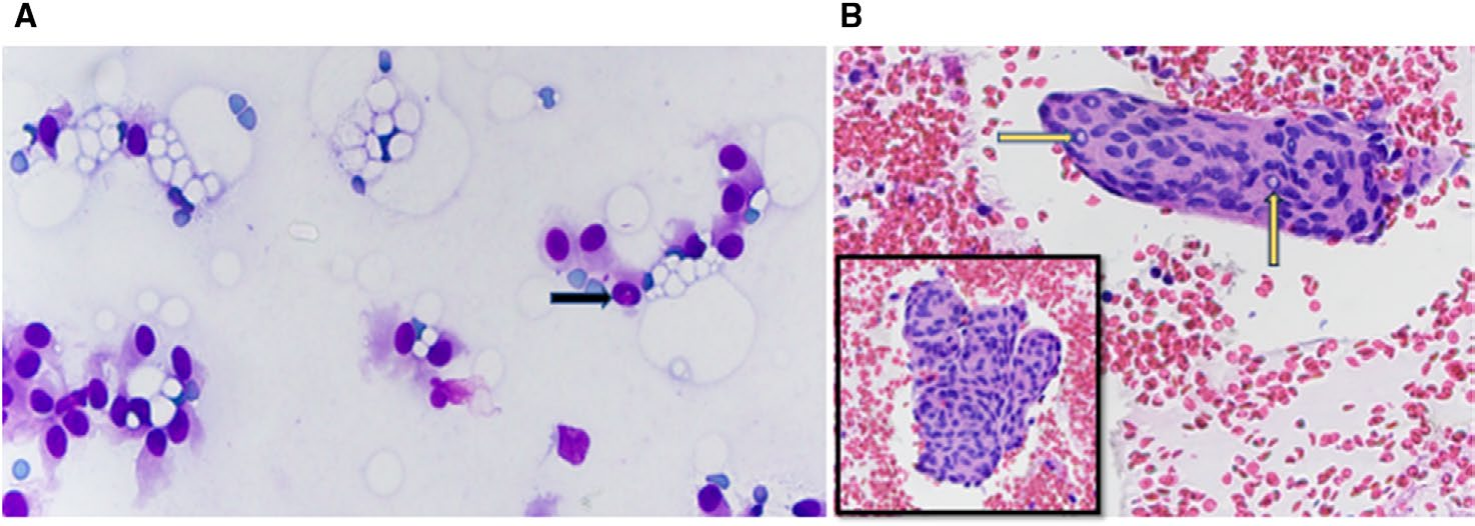
(A) Cytologic smears showing epithelioid cells with wispy cytoplasm, ill defined borders, oval nucleus and an occasional intranuclear pseudoinclusion (black arrow) (DQ, 400×); (B) Cell block demonstrating papillary fragments (inset) with syncytial arrangement of cells having frequent intranuclear pseudoinclusions (yellow arrows) (HE, 400×).

**FIGURE 3:**
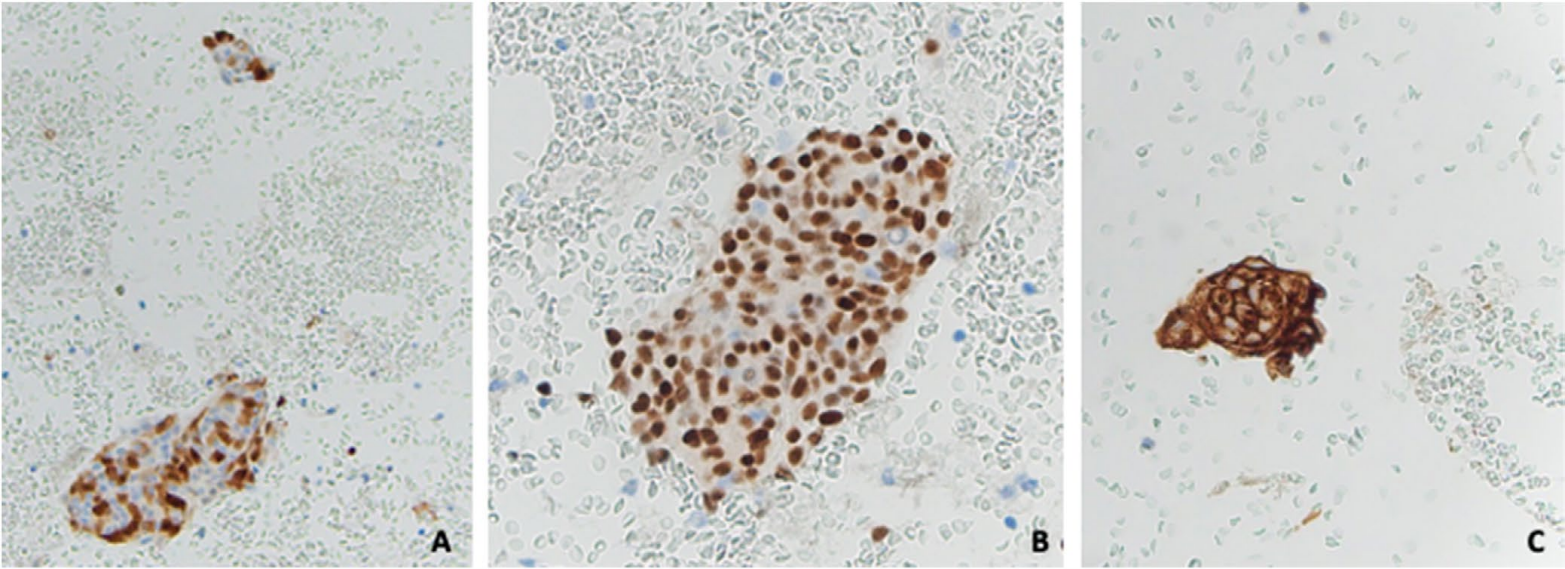
(A) Lesional cell groups showing patchy but strong staining with S‐100 (IHC, 200×); (B) intense nuclear positivity for PR (IHC, 400×); and (C) intense membranous staining for SSTR2A (IHC, 400×).

An ultrasound guided FNA was attempted given the lack of intense vascularity on imaging. Rapid On‐Site Adequacy (ROSE) assessment was inadequate since the Diff Quik stained smears were paucicellular with abundant blood in the background. Four passes were obtained, with two taken directly into formalin for the cell block preparation. Cytologic smears showed sparse groups of epithelioid cells with poorly defined cytoplasmic borders, a moderate amount of pale blue wispy cytoplasm, central oval nuclei with fine chromatin, and occasional intranuclear pseudoinclusions (Figure 2A). The cell block showed a few cell clusters with papillary architecture, a vague whorling pattern, syncytial arrangement of cells, oval regular nuclei with nuclear streaming, and frequent intranuclear pseudoinclusions (Figure 2B). Immunohistochemistry (IHC) was performed, and the lesional cells showed patchy strong nuclear and weak cytoplasmic staining with S‐100 (Figure 3A), but were negative for pancytokeratin, Epithelial Membrane Antigen (EMA) and neuroendocrine markers including synaptophysin and INSM‐1. A second IHC panel, selected based on the morphologic features, revealed that the tumour cells were strongly positive for Progesterone Receptor (PR) (Figure 3B) and Somatostatin Receptor 2A (SSTR2A) consistent with meningothelial differentiation (Figure 3C). Based on the morphology and IHC, a diagnosis of meningothelial meningioma was suggested after intradepartmental consultation.

This case was discussed in tumour board, and the imaging was revisited considering the cytology. The lack of intense arterial enhancement and splaying of the carotid bifurcation, the presence of calcifications near the skull base, and smooth vascular narrowing argued against a paraganglioma. A final integrated cytoradiologic diagnosis of meningioma was rendered, which likely originated from the skull base or the cervical spine and extended into the carotid space. However, there was no obvious evidence of skull base erosion, and a head CT was never performed given the initial radiologic suspicion for a paraganglioma; thus, the exact origin of the tumour could not be defined. The patient was asked to follow up for surgical planning, but she has unfortunately not kept her scheduled appointments with the provider.

## 3 Discussion

Masses detected in and around the carotid artery bifurcation are uncommon and can have a wide spectrum of pathologies. They are commonly diagnosed as extra‐adrenal paragangliomas, benign nerve sheath tumours such as schwannomas, lipomas, pathologies of the cervical lymph node chain, and rarely, cervical extensions of intracranial or dura‐based neoplasms such as meningiomas [1, 2]. Due to the complex anatomy of this region and its intimate relationship with large vascular channels, imaging with contrast enhanced CT (CECT), DSA and/or MRI is the first step in their evaluation. MRI is considered the imaging modality of choice given its high soft tissue resolution and non‐ionising nature; but long imaging wait time, high costs and limited availability often make CECT the first choice for the work‐up of neck masses. Ga68‐Dotatate‐PET CT is complementary to conventional imaging for local staging of paragangliomas and to look for metastatic involvement [1].

Meningiomas with extension into the neck have been reported in literature by at least 9 authors [3]. They are typically seen in women in the 3rd to 5th decade, presenting as slow‐growing masses with neck fullness, hoarseness of voice, or dysphagia due to mass effect. Complete surgical resection is the treatment of choice, with radiotherapy as an adjunct; since all published cases are individual case reports, long‐term follow‐up for these patients was not available in the literature [3].

Imaging guided FNA is of immense help in the precise diagnosis of carotid bifurcation masses. Cytologically, paragangliomas show clusters of moderately pleomorphic cells with eosinophilic granular cytoplasm, round to oval nuclei with stippled chromatin and transgressing vessels with scattered bare nuclei in the background [4, 5]. Lesional cells are strongly positive for synaptophysin, chromogranin, CD56 and INSM‐1, with spindly sustentacular cells staining with S‐100. Meningiomas typically contain round to oval cells in syncytial groups or papillary arrangements with fine nuclear chromatin, abundant intranuclear pseudoinclusions and may show psammomatous calcifications [6, 7]. Meningothelial cells typically stain strongly and diffusely with PR and SSTR2A in ~70% of cases, show strong albeit patchy staining with EMA, and may show patchy S‐100 positivity but are negative for pancytokeratin, desmin and synaptophysin. Diagnosis may be challenging in scantly cellular specimens or those with a predominantly spindly morphology without evidence of pathognomic morphologic features such as calcifications or whorls; SOX‐10 may be helpful here since nerve sheath tumours are typically positive for SOX‐10 while meningiomas are negative. Cell blocks are instrumental in the evaluation of neck masses and even in scenarios like ours with limited tissue quantity, they allow IHC analysis to provide confirmation of diagnosis and appropriate clinical management without the need for repeat sampling or core biopsy.

This case highlights the significance of morphologic and radiologic correlation, especially when it comes to atypical imaging findings of lesions in anatomically risky regions of the head and neck. Fine needle aspiration with cell block preparation is a safe, quick, and valuable modality for morphologic evaluation of such challenging lesions and can help define the underlying pathology by allowing for ancillary workup. A wide range of morphologic differentials must be considered when evaluating carotid body masses since dura‐based neoplasms may rarely extend into the neck, adding to the complexity in diagnosis [3].

## Author Contributions

**Poorva Singh:** conceptualization (lead), writing – original draft (lead), writing – review and editing (lead). **Liam Chen:** writing – review and editing (equal). **Zuzan Cayci:** writing – review and editing (equal), supervision (equal). **Khalid Amin:** writing – original draft (equal), writing – review and editing (equal), supervision (lead).

## Disclosure

Statement of eligibility for the mina desai early career investigator award: POORVA SINGH, the first and corresponding author, is currently a pathology fellow (Molecular Genetic Pathology) and is eligible for this award.

## Conflicts of Interest

The authors declare no conflicts of interest.

## Data Availability

The data that support the findings of this study are available from the corresponding author upon reasonable request.
